# Stereological Investigation of Regional Brain Volumes after Acute and Chronic Cuprizone-Induced Demyelination

**DOI:** 10.3390/cells8091024

**Published:** 2019-09-03

**Authors:** Tanja Hochstrasser, Sebastian Rühling, Kerstin Hecher, Kai H. Fabisch, Uta Chrzanowski, Matthias Brendel, Florian Eckenweber, Christian Sacher, Christoph Schmitz, Markus Kipp

**Affiliations:** 1 Department of Anatomy II, Ludwig-Maximilians-University of Munich, 80336 Munich, Germany (S.R.) (K.H.) (K.H.F.) (U.C.) (C.S.); 2 Institute of Anatomy, Rostock University Medical Center, 18057 Rostock, Germany; 3 Department of Nuclear Medicine, University Hospital, Ludwig-Maximilians-University of Munich, 81377 Munich, Germany (M.B.) (F.E.) (C.S.)

**Keywords:** multiple sclerosis, cuprizone, atrophy, design-based stereology, 18F-FDG

## Abstract

Brain volume measurement is one of the most frequently used biomarkers to establish neuroprotective effects during pre-clinical multiple sclerosis (MS) studies. Furthermore, whole-brain atrophy estimates in MS correlate more robustly with clinical disability than traditional, lesion-based metrics. However, the underlying mechanisms leading to brain atrophy are poorly understood, partly due to the lack of appropriate animal models to study this aspect of the disease. The purpose of this study was to assess brain volumes and neuro-axonal degeneration after acute and chronic cuprizone-induced demyelination. C57BL/6 male mice were intoxicated with cuprizone for up to 12 weeks. Brain volume, as well as total numbers and densities of neurons, were determined using design-based stereology. After five weeks of cuprizone intoxication, despite severe demyelination, brain volumes were not altered at this time point. After 12 weeks of cuprizone intoxication, a significant volume reduction was found in the corpus callosum and diverse subcortical areas, particularly the internal capsule and the thalamus. Thalamic volume loss was accompanied by glucose hypermetabolism, analyzed by [^18^F]-fluoro-2-deoxy-d-glucose (18F-FDG) positron-emission tomography. This study demonstrates region-specific brain atrophy of different subcortical brain regions after chronic cuprizone-induced demyelination. The chronic cuprizone demyelination model in male mice is, thus, a useful tool to study the underlying mechanisms of subcortical brain atrophy and to investigate the effectiveness of therapeutic interventions.

## 1. Introduction

Multiple sclerosis (MS) is a chronic, inflammatory demyelinating disease of the central nervous system. The majority of MS patients experience two clinical phases reflecting distinct but inter-related pathologies. The first phase is characterized by recurrent episodes of immune-driven inflammation and demyelination, from which the patients can recover completely (relapsing–remitting (RR) MS). In the second phase, a continuous progression of clinical, permanent disability can be observed, while the relapse frequency decreases [1,2]. This secondary disease phase is called secondary progressive MS (SPMS), and the findings of several imaging and pathological studies suggest that neuro-axonal damage plays a central role for the observed clinical disease progression [3,4,5]. Current therapeutic strategies for MS are beneficial during RRMS by modulating or suppressing immune function [6]. However, such therapies have no or just a moderate impact on the progressive phase, and therapeutic options effective in SPMS are still unsatisfactory [7].

Much of the research in MS focused on the inflammatory aspects of the disease. As a consequence, the inflammatory component of MS animal models, such as peripheral immune cell recruitment or T-cell-mediated oligodendrocyte pathology, was studied in detail [8,9,10]. In contrast, the neurodegenerative aspect of the disease is by far less frequently addressed and, if so, these studies merely focused on axonal degeneration [11,12]. In particular, brain atrophy, which is a key outcome measure during clinical studies [5,13,14], is almost never evaluated in MS animal models, although magnetic resonance imaging (MRI)-based protocols were published [15,16]. In MS patients, MRI measurements revealed white-matter lesions, as well as gray-matter atrophy of specific brain areas including cortical and subcortical gray matter [17,18,19]. The extent of atrophy was found to be more severe in the subcortex compared to the superficial cortex, and was found to correlate with cognitive performance and clinical disability in MS patients [19,20]. Thus, establishing the histological correlate of volumetric changes in the brain is of significant importance [21,22].

The cuprizone model is a well-established mouse model of MS. Following oral administration of the copper-chelating agent cuprizone, the mouse brain exhibits a variety of pathological alterations including demyelination of the white and gray matter, gliosis, and axonal damage [23,24]. While acute demyelination is usually induced by five weeks of cuprizone feeding, sustained (chronic) demyelination can be achieved by feeding mice with cuprizone for 12 weeks. Although axonal damage is found after acute and chronic demyelination in this model [11,12,25], there is little data available on neuronal loss and volumetric changes in the different vulnerable brain regions. Previous imaging studies revealed volumetric changes in the corpus callosum and the cortex after cuprizone intoxication [26,27,28]. Furthermore, histopathological studies showed neuronal pathologies, including loss of neurons in the hilus of the dentate gyrus, as well as loss of parvalbumin inhibitory interneurons in the hippocampus CA1 subregion of chronically demyelinated mice [29,30]. However, the extent of cortical and subcortical atrophy determined by standardized histological techniques (i.e., design-based stereology [31]) is still unexplored; therefore, the significance of this model to study the underlying mechanisms of brain atrophy in MS is unknown.

Thus, in the present study, we used design-based stereology to analyze callosal, cortical, and subcortical volumes, as well as neuron numbers and densities after acute and chronic cuprizone-induced demyelination.

## 2. Materials and Methods

### 2.1. Animals

Male C57BL/6J mice were purchased from Janvier (Le Genest-Saint-Isle, France), and were housed in a temperature-controlled environment (21–24 °C) with humidity levels between 55% and 65% on a 12-h light/dark cycle. Chow and water were provided ad libitum. All experiments were performed according to the Federation of European Laboratory Animal Science Association recommendations and were approved by the Review Board for the Care of Animal Subjects of the district government of Upper Bavaria (55.2-1-54-2532-73-15).

### 2.2. Cuprizone Intoxication and Tissue Processing

To induce acute or chronic demyelination, male mice (19–21g) were intoxicated with 0.25% cuprizone (bis(cyclohexanone)oxaldihydrazone, Sigma-Aldrich, St. Louis, MO, USA) mixed into ground standard rodent chow (Ssniff, Soest, Germany) for five or 12 weeks, respectively. Control groups were fed with cuprizone-free standard rodent chow. Detailed numbers of animals used for the different experiments are provided in the figure legends. After five or 12 weeks, the animals were perfused with ice-cold phosphate-buffered saline (PBS) followed by a 3.7% formaldehyde solution (pH 7.4). After overnight post-fixation in the same fixative, brains were carefully removed from the skull and processed for paraffin embedding or cryoprotection following established protocols [12,32]. For the preparation of paraffin sections, the brains were dehydrated, cleared with xylene, embedded in paraffin, and cut into 5-µm-thick coronal sections using a sliding microtome (Type SM 2000R; Leica Microsystems, Wetzlar, Germany). To prepare cryo-sections, post-fixed brains were rinsed in PBS and cryoprotected in sucrose solutions (10%, 20%, and finally 30% sucrose (*w*/*v*) in PBS) at 4 °C for 24 h in each solution. Thereafter, brains were frozen in isopentane (−70 °C, 1 min) on dry ice, cut into 40-µm-thick coronal serial sections on a cryostat (Type CM 1950; Leica Microsystems), and stored at −20 °C in a cryoprotective solution (30% ethylene glycol, 30% glycerol in PBS) until further processing. 

For immunohistochemical analyses of paraffin sections, two consecutive sections (level R265 according to Reference [33]) were processed as previously described [34]. In brief, sections were deparaffinized, rehydrated, heat-unmasked by Tris–ethylene diamine tetraacetic acid (EDTA) (pH 9.0) and blocked in 5% normal goat serum or a mixture of 2% normal goat serum, 0.1% cold water fish skin gelatin, 1% bovine serum albumin, and 0.05% Tween-20. Sections were incubated with the following primary antibodies overnight at 4 °C: myelin proteolipid protein (PLP), 1:5000, RRID:AB_2237198, Bio-Rad, Hercules, CA, USA; ionized calcium-binding adapter molecule 1 (IBA1), 1:5000, RRID:AB_2665520, Wako, Neuss, Germany; amyloid precursor protein (APP), 1:5000, RRID:AB_94882, Merck-Millipore, Burlington, VT, USA. On the next day, sections were treated with 0.3% hydrogen peroxide in PBS, and the biotinylated secondary antibodies (goat anti-mouse immunoglobulin G (IgG), goat anti-rabbit IgG, 1:200; Vector labs, Burlingame, CA, USA) were applied for 1 h. Thereafter, sections were incubated in peroxidase-coupled avidin–biotin reagent (ABC kit, Vector labs) and the antigen–antibody complexes were finally visualized by 3,3′-diaminobenzidine (Dako, Hamburg, Germany). Sections were counterstained with standard hematoxylin to visualize cell nuclei if appropriate.

**Figure 1:**
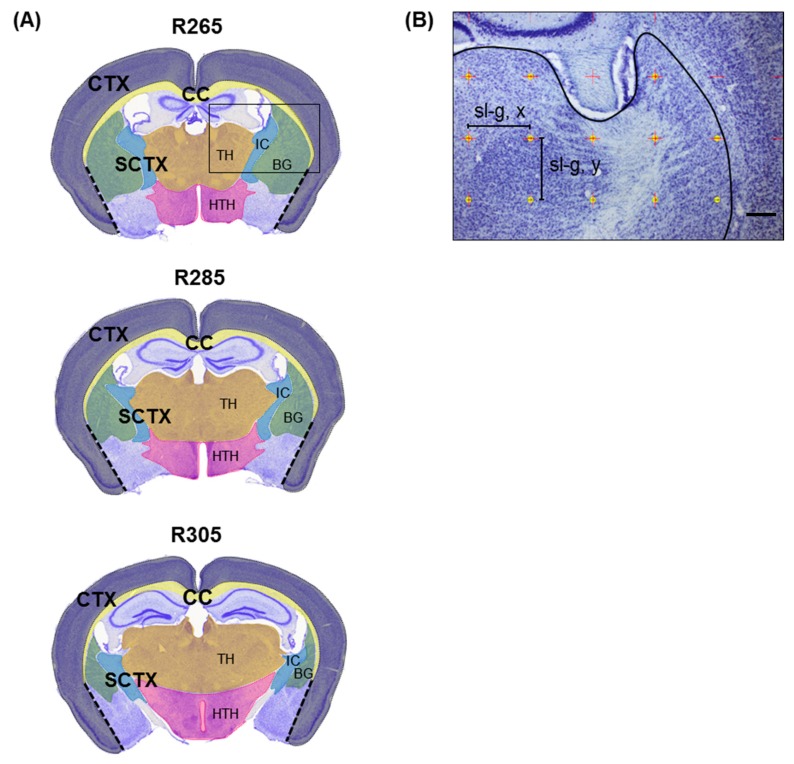
(**A**) Representative coronal sections of a mouse brain at levels R265, R285, and R305 (according to Reference [33]) showing regions investigated in the present study (corpus callosum (CC, yellow); cerebral cortex (CTX, gray); thalamus (TH, brown); hypothalamus (HTH, pink); internal capsule (IC, blue); basal ganglia (BG, green)). (**B**) Representative photomicrograph of a Nissl-stained coronal section (enlarged view of black square in (**A**)). The slide is superimposed with a rectangular grid (intersections are represented by red crosses; sl-g = side length in *x-* and *y*-directions of the grid) to estimate the volume of the designated areas. Grid intersections within the subcortical area are highlighted as yellow dots. Scale bar in (**B**) = 1 mm (**A**) and 200 µm (**B**).

For stereological analyses of cryostat sections, every third section between levels R265 and R305 (according to Reference [33]; Figure 1) was selected with a random start (first series). In these regions, demyelination can be induced in a highly reproducible manner in the corpus callosum [35,36]. Demyelination is not only restricted to this white-matter tract but also involves other brain areas including the cerebral cortex, basal ganglia, and thalamus [23,37,38]. Sections were mounted on gelatinized glass slides, dried, and stained with cresyl violet (Nissl staining) [39]. For a second series of sections (every sixth section), free-floating immunofluorescence staining was performed. Sections were blocked in blocking solution (10% normal donkey serum/0.5% Triton X-100/PBS) and incubated with the primary antibodies (NeuN, 1:2000, RRID:AB_2298772, Merck-Millipore) for 48 h at 4 °C. Thereafter, sections were incubated with the secondary antibodies (donkey anti-mouse Alexa Fluor 488, 1:500; Life Technologies, Carlsbad, CA, USA) for 6 h. Cell nuclei were counterstained using DAPI (4,6-diamidino-2-phenyl-indole; Life Technologies), and sections were mounted on gelatinized glass slides and coverslipped using FluorPreserve reagent (Merck-Millipore).

### 2.3. Evaluation of Histological Parameters and Stereological Analysis

To validate demyelination and microgliosis in the cuprizone model, two consecutive (PLP- and IBA1-stained) sections per mouse were evaluated by densitometry of the staining intensity, and the results were averaged. Staining intensity of the region of interest (ROI) was quantified using ImageJ (NIH, version 1.47v, Bethesda, MD, USA) and is given as percentage myelination or percentage area of the entire ROI. For quantification of axonal damage, APP^+^ spheroids were counted as previously described [23]. In general, densities are given in spheroids or cells per square millimeter (mm^2^). 

Stereological analyses were performed with the stereology software (Stereo Investigator, version 11.07; MBF Bioscience, Williston, ND, USA) and either a light microscope (Olympus BX51WI; Olympus, Tokyo, Japan) equipped with a motorized specimen stage (MBF Bioscience), UPlanApo objective (4×, numerical aperture (N.A.) = 0.16; Olympus), and a charge-coupled device (CCD) color video camera (U-CMAD-2; Olympus) or a modified fluorescence microscope (Olympus BX51; Olympus) equipped with a motorized specimen stage (MBF Bioscience), a customized spinning disk unit (DSU; Olympus), UPlanSApo objective (20×, N.A. = 0.75; Olympus), Alexa Fluor 488 filter (excitation: 498 nm, emission: 520 nm; Chroma, Bellows Falls, VT, USA), and a Retiga 2000R CCD camera (Q-Imaging, Surrey, BC, Canada).

The first series of sections was stained with cresyl violet to analyze the volumes of the selected brain regions. The volumes of the distinct brain regions (cerebral cortex, subcortical area, corpus callosum, internal capsule, hypothalamus, thalamus, and basal ganglia (globus pallidus and caudoputamen)) were calculated using Cavalieri’s principle [40]. Areas of brain regions were determined by tracing their boundaries on each section using the stereology software. Medial boundaries of the cortex (CTX) were defined by the corpus callosum and a line drawn between the basal tip of the corpus callosum and the basal end of the piriform area (shown in Figure 1A as dashed line). The thalamus, hypothalamus, basal ganglia, and internal capsule were summed up as the subcortical area (SCTX). To estimate the volume of the distinct brain regions, the slides were superimposed by a rectangular grid (Figure 1B, Table 1). Then, the volumes of brain regions were calculated by summing the counted points (grid intersections) from all sections (Table 1) and multiplying this value with the area associated with each point, the section interval, and the average cut section thickness. 

The second series of sections was stained with anti-NeuN antibodies to analyze total neuronal numbers and densities within the distinct brain regions using the optical fractionator method [40,41]. The base of the unbiased virtual counting spaces (UVCS) was 1225 µm^2^, the height was 15 µm, and the upper guard zone was 4 µm. The distance between the UVCS in *x*- and *y*-directions was 1100 µm and 1100 µm, respectively (Table 1). All neurons whose nucleus top came into focus within the UVCS were counted. Then, total neuronal numbers were calculated from the numbers of marked neurons (Table 1) and the corresponding sampling probability. Neuronal densities were calculated as the ratio of total numbers of neurons and the volume (measured section thickness × sum of all cross-sectional areas) of the region. The fractional volumes occupied by NeuN immunoreactive perikarya or the neuropil in the SCTX were estimated by counting randomly positioned test points (grid intersections) falling on nerve cell perikarya or the neuropil according to Cavalieri’s principle.

### 2.4. [^18^F]-Fluoro-2-deoxy-d-glucose Positron-Emission Tomography (FDG PET) Imaging

Chronic cuprizone-intoxicated mice (12 weeks of cuprizone) and controls were imaged with [^18^F]-FDG-PET on the last day of treatment. Then, µPET imaging followed a standardized protocol [42]. In brief, 12.4 ± 2.1 MBq [^18^F]-FDG was injected into the tail vein after a fasting period >3 h. Emission was acquired from 30 to 60 min post injection followed by a 15-min transmission scan. All images were co-registered to a [^18^F]-FDG-PET mouse template and normalized to standardized uptake values (SUV). Predefined brain regions of the Mirrione mouse atlas implemented in PMOD v3.5 (PMOD technologies, Basel, Switzerland) were applied to extract individual regional glucose metabolism of all studied mice. Averaged SUV images of chronic cuprizone-intoxicated mice and control mice were used to calculate voxel-based percentage changes between both groups. 

### 2.5. Statistical Analyses

For each group of animals, data are presented as individual values and means per group. Data were firstly analyzed using the Kolmogorov–Smirnov test for normality. Differences between groups were then analyzed using Student’s *t*-test or the Mann–Whitney U test, as appropriate. Data were not corrected for multiple comparisons. The *p*-values and the applied statistical procedures are given in the respective figure legends. A *p*-value <0.05 was considered to be statistically significant. Calculations were performed using GraphPad Prism (GraphPad Software Inc., version 5.04; San Diego, CA, USA). 

## 3. Results

### 3.1. Acute Demyelination Does Not Lead to Brain Volume Loss

**Figure 2:**
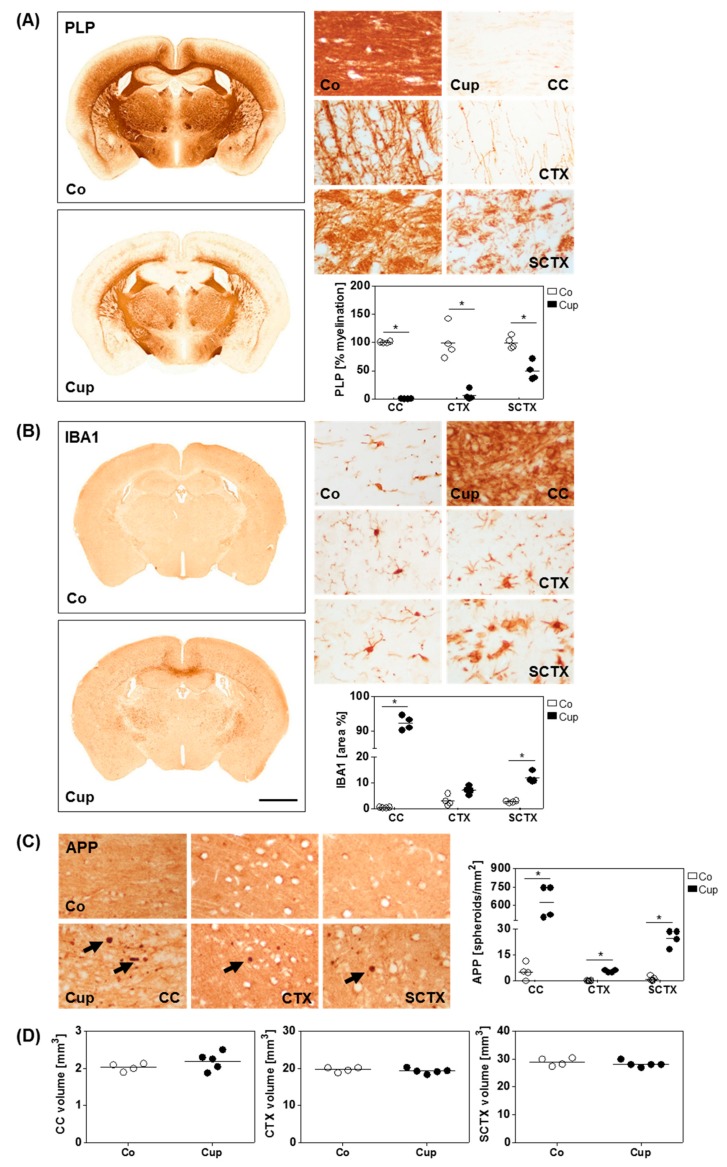
Acute demyelination does not lead to volumetric changes. (**A**) Quantification of myelination (anti-myelin proteolipid protein (PLP)) and (**B**) microgliosis (anti-ionized calcium-binding adapter molecule 1 (IBA1)) from controls (Co) (*n* = 4) and five weeks of cuprizone-intoxicated (Cup) mice (*n* = 4). Individual brain regions are shown in higher magnification: medial corpus callosum (CC), cortex (CTX), and subcortical area (SCTX). (**C**) Quantification of axonal damage (anti-amyloid precursor protein (APP)) with representative pictures of the medial CC, CTX, and SCTX from Co and Cup mice. Arrows indicate APP^+^ spheroids. (**D**) Volumes of the CC, CTX, and the SCTX of Co (*n* = 4) and Cup mice (*n* = 5), analyzed with design-based stereology. Data are shown as individual values and means per group (lines). Statistical analysis revealed significant differences between Co and Cup in (**A**) (CC, *p* = 0.03, CTX, *p* = 0.03, SCTX, *p* = 0.03), (**B**) (CC, *p* = 0.03; SCTX, *p* = 0.03), and (**C**) (CC, *p* = 0.02, CTX, *p* = 0.02, SCTX, *p* = 0.02); *p < 0.05. Scale bar in (**B**) = 2 mm (**A**,**B**) and 17 µm (enlarged views (**A**,**B**)) and (C).

We firstly investigated the severity of acute cuprizone-induced injury (i.e., demyelination, microglia activation, and axonal damage) in the different brain regions. To this end, animals were intoxicated with cuprizone for five weeks, and myelination (anti-PLP), microgliosis (anti-IBA1), and acute axonal damage (anti-APP) were compared to controls which were fed normal chow during the experimental period. In line with previous findings [23,34,36,43,44], immunohistochemical analyses revealed extensive demyelination of the medial corpus callosum, the cerebral cortex, and the subcortical area after five weeks of cuprizone intoxication (Figure 2A). Demyelination was most severe in the corpus callosum, followed by the cerebral cortex and the subcortical area. In the corpus callosum and the subcortical area, acute demyelination was paralleled by increased microglia densities (Figure 2B), in line with previous results from our group [23,36]. In the cerebral cortex, microglia activation was less severe, but IBA1^+^ cells clearly showed an activated phenotype (swollen cell bodies and retracted processes; see Figure 2B). To examine the extent of acute axonal damage, we quantified APP^+^ spheroids, a marker of acute axonal injury. As shown in Figure 2C, numerous APP^+^-spheroids were found in the corpus callosum, the cerebral cortex, and the subcortical area of cuprizone-intoxicated animals. Such spheroids were not observed in control animals. 

Furthermore, we investigated whether acute cuprizone-induced injury results in overall brain volume changes in the analyzed region (R265–R305). Mean overall volume was not altered in cuprizone-intoxicated versus control mice (control (Co): 63.68 mm^3^ ± 1.15 mm^3^; cuprizone (Cup): 64.34 mm^3^ ± 0.89 mm^3^; *p* = 0.076). Furthermore, we measured the volume of the corpus callosum, the cerebral cortex, and the subcortical area by stereological analysis. Despite extensive demyelination and axonal damage, individual brain volumes for the corpus callosum, cortex, and subcortical area were comparable in control and five weeks of cuprizone-intoxicated mouse brains (Figure 2D). 

### 3.2. Chronic Demyelination Results in Regional Brain Volume Loss

**Figure 3:**
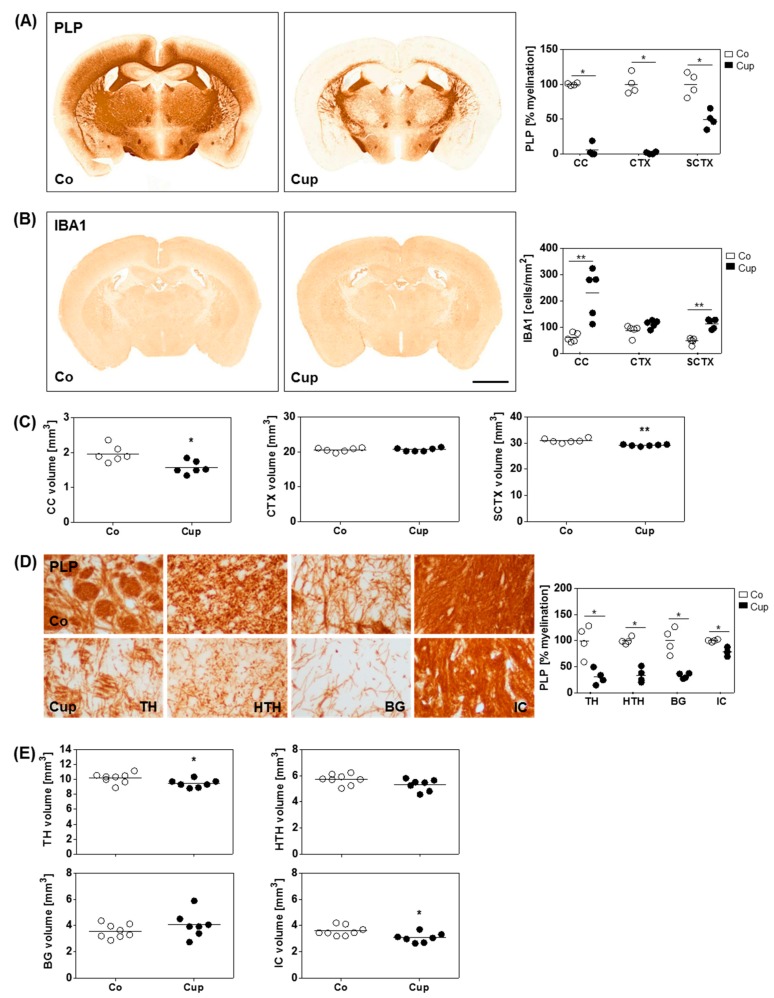
Chronic demyelination leads to subcortical volumetric reduction. (**A**) Quantification of myelination (anti-PLP) and (**B**) microgliosis (anti-IBA1) in the medial corpus callosum (CC), cortex (CTX), and subcortical area (SCTX) from controls (Co) (*n* = 4) and five weeks of cuprizone-intoxicated (Cup) mice (*n* = 5). (**C**) Volumes of the CC, CTX, and the SCTX of Co (*n* = 6) and Cup mice (*n* = 6) analyzed with design-based stereology. (**D**) Quantification of myelination with representative pictures of individual subcortical regions (thalamus (TH), hypothalamus (HTH), basal ganglia (BG), internal capsule (IC)) from Co (*n* = 4) and Cup mice (*n* = 4). (**E**) Volumes of the TH, HTH, STR, and IC of Co (*n* = 8) and Cup mice (*n* = 7). Data are shown as individual values and means per group (lines). Statistical analysis revealed significant differences between Co and Cup in (**A)** (CC, *p* = 0.03, CTX, *p* = 0.03, SCTX, *p* = 0.03), (**B)** (CC, *p* = 0.008; SCTX, *p* = 0.008), (**C**) (CC, *p* = 0.02; SCTX, *p* = 0.0012), (**D**) (TH, *p* = 0.03; HTH, *p* = 0.03; BG, *p* = 0.03; IC, *p* = 0.03), and **E** (TH, *p* = 0.04; IC, *p* = 0.02); *p <0.05, **p < 0.01. Scale bar in (**B**) = 2 mm (**A**,**B**) and 17 µm (**D**).

In a next step, we investigated histopathological changes after chronic cuprizone intoxication. Therefore, mice were fed with cuprizone for 12 weeks, and myelination and microglia activation were evaluated. As shown in Figure 3A, severe loss of anti-PLP staining intensity was evident in several brain regions, including the corpus callosum and cerebral cortex of animals intoxicated for 12 weeks. Moreover, moderate but consistent demyelination was observed in the subcortical area. Of note, chronic demyelination was paralleled by increased microglia densities (Figure 3B). To analyze volumetric changes after chronic demyelination, we analyzed the overall brain volume (R265–R305). The overall volume of cuprizone-intoxicated mice was significantly smaller compared to control mice (Co: 67.16 mm^3^ ± 0.66 mm^3^; Cup: 65.28 mm^3^ ± 0.38 mm^3^; *p* = 0.045). Next, we measured the individual brain volumes after chronic cuprizone-induced demyelination. Mean volumes of the corpus callosum and the subcortical area were significantly smaller in cuprizone-intoxicated versus control mice. In particular, the volume of the corpus callosum was 1.96 mm^3^ ± 0.10 mm^3^ in control, and 1.59 mm^3^ ± 0.09 mm^3^ in cuprizone-intoxicated mice (*p* = 0.02), whereas the volume of the subcortical area was 30.96 mm^3^ ± 0.34 mm^3^ in control, and 29.11 mm^3^ ± 0.14 mm^3^ in cuprizone-intoxicated mice (*p* = 0.0012). No volume loss was observed in the cortex (Figure 3C). 

As demonstrated above, a volume reduction after chronic cuprizone-induced demyelination was found in the subcortical area, which consists of distinct white- and gray-matter areas including different functional nuclei. Next, we were interested which subcortical region is most vulnerable in the cuprizone model. Thus, we decided to separately evaluate the volumes of the thalamus, hypothalamus, basal ganglia, and the internal capsule, and contrast atrophy with the extent of demyelination. As demonstrated in Figure 3D, anti-PLP staining intensities were clearly reduced in the thalamus, hypothalamus, and basal ganglia, yet demyelination was incomplete. In contrast, loss of anti-PLP staining intensity was moderate in the internal capsule (Figure 3D). Volumetric measurements revealed that the mean volumes of the internal capsule and the thalamus were significantly lower in cuprizone-intoxicated versus control mice. In particular, the volume of the internal capsule was 3.60 mm^3^ ± 0.14 mm^3^ in control, and 3.08 mm^3^ ± 0.14 mm^3^ in cuprizone-intoxicated mice (*p* = 0.04), whereas the volume of the thalamus was 10.18 mm^3^ ± 0.24 mm^3^ in control, and 9.45 mm^3^ ± 0.20 mm^3^ in cuprizone-intoxicated mice (*p* = 0.02). In contrast, the mean hypothalamus and basal ganglia volumes were not found to be reduced after chronic demyelination (Figure 3E).

### 3.3. Axonal Damage Rather Than Neuronal Loss Contributes to Brain Volume Loss

**Figure 4:**
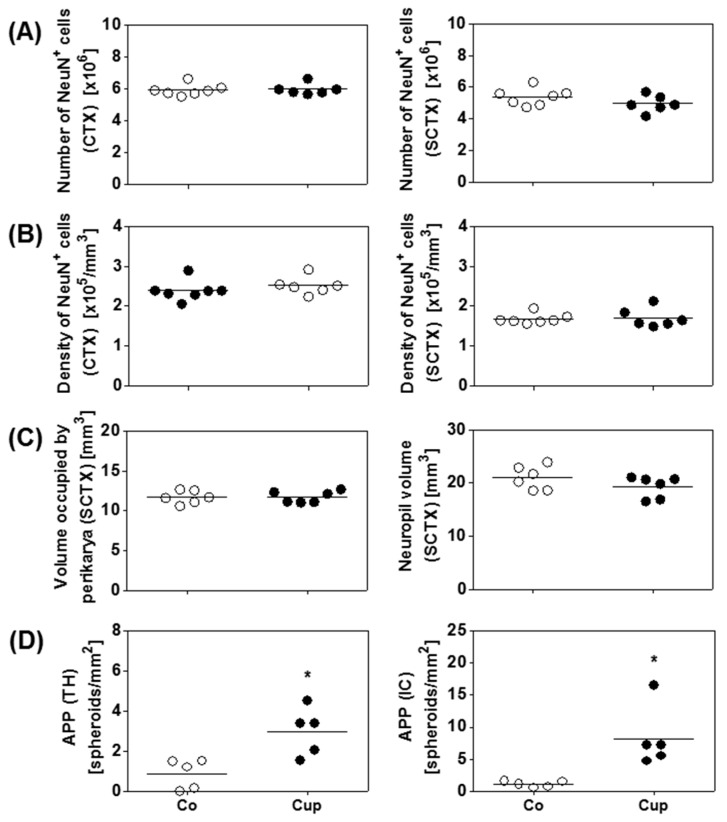
Effects of chronic demyelination on the neuro-axonal/-dendritic compartment. (**A**) Numbers and (**B**) densities of neurons in the cerebral cortex (CTX) and the subcortical area (SCTX) of control (Co) (*n* = 6) and 12 weeks of cuprizone-intoxicated (Cup) mice (*n* = 6) analyzed with design-based stereology. (**C**) Neuropil volumes and volumes occupied by nerve cell perikarya in the SCTX of Co (*n* = 6) and 12-week Cup mice (*n* = 6). (**D**) Quantification of amyloid precursor protein (APP)-positive spheroids in the thalamus (TH) and internal capsule (IC) of chronically demyelinated mice (*n* = 5). Data are shown as individual values and means per group (lines). Statistical analysis revealed significant differences between Co and Cup in (**D**) (TH, *p* = 0.02; IC, *p* = 0.02); *p < 0.05.

To investigate whether the observed subcortical volume reduction results from (i) neuronal cell body loss and/or (ii) acute axonal damage, we firstly quantified the mean numbers of neurons and mean densities of neurons in the cerebral cortex and the entire subcortical area using design-based stereology. In the cerebral cortex, no alterations in mean neuronal numbers and mean neuronal densities were detected in 12 weeks of cuprizone-intoxicated mice (Figure 4A,B). Of note, also in the subcortical area, the mean numbers and densities of neurons were comparable in 12 weeks of cuprizone-intoxicated mice as compared to age-matched controls (Figure 4A,B). Furthermore, in the entire subcortical area, the space occupied by perikarya and the neuropil was not significantly different in cuprizone-intoxicated versus control mice (Figure 4C). 

Secondly, we investigated the densities of APP^+^ spheroids. We focused in this part of the study on the thalamus and the internal capsule because volume loss was found to be most severe in these two subcortical brain areas. As shown in Figure 4D, numerous APP^+^ spheroids were found in the thalamus and the internal capsule of 12 weeks of cuprizone-intoxicated mice. Such spheroids were not observed in control animals (Figure 4D).

### 3.4. Mice with Chronic Demyelination Show Higher Uptake of [^18^F]-FDG in the Thalamus

**Figure 5:**
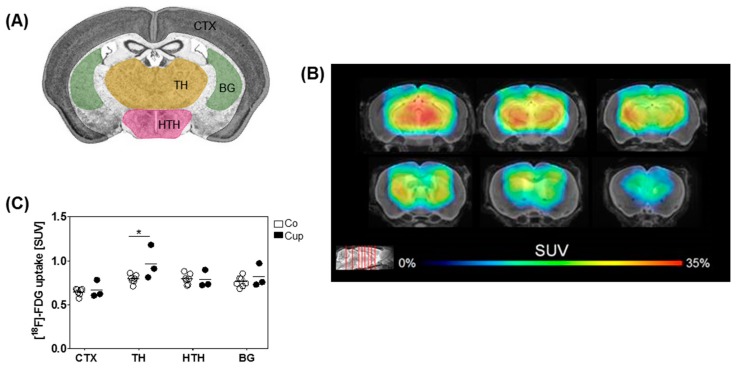
Altered [^18^F]-fluoro-2-deoxy-d-glucose ([^18^F]-FDG) ligand uptake after chronic cuprizone intoxication. (**A**) Schematic illustration of the different brain regions (cortex (CTX), thalamus (TH), hypothalamus (HTH), and basal ganglia (BG)), evaluated for [^18^F]-FDG ligand uptake. (**B**) Cumulative heat map illustrating percentage increases of standardized uptake values (SUV) in cuprizone-treated mice relative to controls. (**C**) Quantification of the normalized [^18^F]-FDG uptake in Co (*n* = 7) and 12-week Cup mice (*n* = 3). Data are shown as individual values and means per group (lines). Statistical analysis revealed significant differences between Co and Cup in (**C**) (TH, *p* = 0.03); *p < 0.05.

Neuroimaging is a central part of the diagnostic work-up of patients with suspected neurodegenerative disease, including MS. [^18^F]-FDG PET is an attractive tool to study neurodegeneration on the metabolic level. Therefore, we were interested whether we could detect metabolic alterations after chronic demyelination. To this end, glucose metabolism in the mouse brain was analyzed using [^18^F]-FDG μPET. Interestingly, we found a region-dependent increase of [^18^F]-FDG uptake in cuprizone-treated mice. A visual interpretation of the [^18^F]-FDG µPET scans indicated increased [^18^F]-FDG µPET signal, particularly in the subcortical area (Figure 5A,B). A statistical comparison between predefined brain regions in the control and cuprizone-intoxicated mice showed significantly higher [^18^F]-FDG uptake in the thalamus of 12 weeks of cuprizone-intoxicated mice (increase of 22%). There were no significant differences in [^18^F]-FDG uptake in the cortex, hypothalamus, and basal ganglia in 12 weeks of cuprizone-intoxicated mice compared to age-matched controls (Figure 5C). 

## 4. Discussion

This is the first study focusing on volume, neuronal densities, and total neuronal numbers in the cerebral cortex and subcortical area after acute and chronic cuprizone-induced demyelination. We could show that specific subregions display volume loss after chronic cuprizone-induced demyelination, particularly the corpus callosum, internal capsule, and the thalamus. Of note, volumetric changes were not paralleled by loss of NeuN^+^ neuronal cell numbers or shrinkage of their perikarya, but by ongoing degeneration of axons, as demonstrated by the ongoing axonal transport deficit of APP. 

Of note is the specific volume loss of the subcortex, whereas no significant volumetric changes were observed in the cerebral cortex. It is well known that, in addition to the corpus callosum, the cortex is a vulnerable brain region in the cuprizone model, demonstrating severe and almost complete demyelination after a five-week cuprizone intoxication period [37,45]. It was, therefore, somewhat surprising that we did not detect cortical atrophy in the cortex even after chronic cuprizone exposure. Since we only evaluated the entire cortex, we cannot rule out that specific cortical subregions, such as the somatosensory and somatomotor cortex, which are particularly vulnerable in the cuprizone model, show reduced volumes [24]. While axonal damage/injury was observed by many groups in the cuprizone model [11,12,30], a decrease in neuronal densities or neuronal cell numbers is less well appreciated. While Tiwari-Woodruff’s lab demonstrated a loss of parvalbumin-expressing interneurons in the hippocampal CA1 region [29], Löscher’s lab showed a loss of neurons in the hilus of the dentate gyrus [30]. While neuronal cell loss in the MS brain clearly exists [46], it is unclear which mechanisms trigger neurodegeneration. In the cuprizone model, demyelination alone seems not to be sufficient to trigger neuronal cell loss, at least in the investigated brain areas. Interestingly, recent post-mortem studies did not reveal any association between the extent of demyelination and the density of neurons in the MS neocortex [47,48]. We speculate that, in addition to metabolic stressors, auto-immune driven inflammatory mediators are necessary to induce neuronal cell loss in the neocortex and, consequently, cortical brain atrophy. A recently described novel MS animal model principally allows studying the interplay of innate and adaptive immunity [49,50,51], and we are currently investigating in ongoing studies the extent to which the liaison of metabolic and inflammatory brain injury triggers neuronal degeneration. 

We are well aware that the underlying mechanisms of MS and cuprizone-induced injury are most likely not the same. Nevertheless, several important aspects of the MS pathology are nicely recapitulated by the cuprizone model. For example, mitochondrial density is increased within demyelinated axons in active MS [52] and cuprizone-induced lesions [53]. Furthermore, splitting of the inner myelin lamella, referred as dying-back oligodendrogliopathy, was reported in MS [54] and cuprizone-induced demyelination [55]. Finally, the presence of apoptotic oligodendrocytes, which express active caspase-3 during lesion formation, was described in MS [56] and the cuprizone model [57]. Thus, understanding mechanisms operant during cuprizone-induced demyelination might help to understand disease mechanisms in MS. 

As pointed out above, significant brain volume loss was found in subcortical regions among the thalamus (see Figure 3E). The thalamus forms the largest part of the diencephalon and is eponymous for other diencephalic components such as the epithalamus and hypothalamus. This diencephalic brain region is highly interconnected with various cortical and subcortical areas. We speculate that the thalamus with its multiple reciprocal connections is sensitive to pathological processes occurring in different brain regions, thus acting as a “barometer” for diffuse brain parenchymal damage in MS [58]. Our findings are in line with a specific subcortical gray-matter atrophy found in MS patients [19,20,59,60]. In these MRI studies, subcortical gray-matter atrophy was most prominent in the thalamus, showing a volume reduction of 12–25%. From a functional point of view, it is interesting to note that the thalamus volume loss strongly correlated to patients’ declining cognitive functions and physical disability [19,20,61,62], highlighting the thalamus as a central structure in the context of MS-related disability and disease progression.

Thalamic volume loss was found to be related to neuro-axonal pathology. Cifelli et al. found a reduction in both neuronal densities and *N*-acetylaspartate (NAA) concentration in the thalamus of MS patients [60]. In the present study, cuprizone-induced demyelination neither affected NeuN^+^ neuronal numbers and densities nor the space occupied by perikarya. Noteworthy, our [^18^F]-FDG-PET measurements indicated a significant hypermetabolism in the thalamus of cuprizone-intoxicated mice when compared to controls, fitting to preserved neuronal numbers in this brain region, as the vast majority of the [^18^F]-FDG-PET signal is supposed to be related to neuronal energy metabolism [63]. Thus, it seems likely that equal neuronal numbers, together with increased glial activity after chronic cuprizone intoxication, will result in higher net energy consumption when compared to controls. Of note, there is still no evidence how much glial cells contribute to the cerebral energy consumption, although it is assumed that glucose metabolism decreases when microglia are dysfunctional [64]. 

Surprisingly, we did not find callosal volume loss after acute cuprizone-induced demyelination, despite significant axonal injury. This could have happened for a number of reasons. Firstly, in our study, we did not measure irreversible axonal degeneration or axonal loss, but rather a deficit of the anterograde axonal transport machinery. The gold-standard method to detect axonal loss is electron microscopy or thin plastic sections. Most previous studies which analyzed acute axonal injury in the cuprizone model visualized either axonal transport deficits or the expression changes of neurofilament proteins [11,65], which does not necessarily represent the true extent of irreversible axonal degeneration [66]. Secondly, due to the severe astrocytosis and microgliosis accompanying the cuprizone-induced demyelination, callosal volume loss might be “masked” despite axonal loss (if present). Thirdly, cuprizone intoxication results in spongy degeneration of the white matter [67], another pathological feature which might mask callosal volume loss. In MS, it was suggested that untreated inflammation and edema might increase the brain volume, leading to an underestimation of true tissue loss. Interestingly, anti-inflammatory therapies were associated with acceleration of brain volume loss, referred to as pseudoatrophy [68]. This is likely caused by the resolution of inflammation and edema rather than true brain atrophy. In our study, chronic cuprizone-induced demyelination did lead to a brain volume reduction despite ongoing microgliosis, suggesting true tissue loss. Furthermore, it was suggested that pseudoatrophy effects are greater in white-matter structures due to larger glial infiltration and activation compared to gray-matter structures. Thus, volume measurement of gray-matter structures might be more reliable to distinguish irreversible changes due to tissue loss from the reversible changes due to pseudoatrophy. The pathological substrate of subcortical volume loss in the cuprizone model is currently unknown. In principle, volume loss might be due to loss of the myelin sheath (i.e., demyelination), glia degeneration, neuronal cell loss, axonal degeneration, or synaptic pathology. Of note, in a post mortem analysis, widespread loss of dendritic spines was found in the cortex of MS patients. Dendritic spine loss occurred in demyelinated and non-demyelinated cortex regions, and preceded loss of cortical axons [69]. In this study, we could show that subcortical volume loss is not paralleled by a reduction of total neuronal cell numbers. However, subcortical neuropil volumes tended to decrease, suggesting degeneration in the axonal and/or dendritic compartment. Of note, in this study, we did not quantify the loss of cell types other than neurons, such as astrocytes or oligodendrocytes, by means of design-based stereology. It, thus, might well be that the observed atrophy in the thalamus, for example, is due to oligodendrocyte or astrocyte degeneration. In a recent study, we were able to show that astrocytes express the stress-associated transcription factor DNA damage-inducible transcript 3 after acute cuprizone-induced demyelination [32]. Further studies are needed to investigate this aspect in more detail.

As already pointed out above, the thalamus shares broad reciprocal connections with other brain regions, such as the cerebral cortex. These connections run within the internal capsule. In the present study, we observed volume reduction and axonal damage of the internal capsule, despite moderate demyelination. Thus, volume loss of the internal capsule might be due to the degeneration of efferent thalamic fiber tracts. This is consistent with findings demonstrating reduced NAA levels in normal-appearing white matter of the internal capsule in MS patients [70]. 

In summary, this study pointed out that the chronic, but not the acute cuprizone model may serve as a valuable tool to study subcortical brain volume loss. Further studies are now warranted to investigate which mechanisms are operant and how this brain volume loss can be ameliorated by pharmacological intervention.

## Figures and Tables

**Table 1:** Details of the stereological estimation procedure.

	CC	CTX	SCTX	TH	HTH	BG	IC
sl-g (µm)	220	500	500	120	120	120	120
∑points_area_	337	669	985	5693	3192	2205	1943
sl-uvf (µm)	-	35	35	-	-	-	-
B (µm^2^)	-	1225	1225	-	-	-	-
H (µm)	-	15	15	-	-	-	-
D (µm)	-	1100	1100	-	-	-	-
∑UVCS	-	155	185	-	-	-	-
∑neurons	-	653	555	-	-	-	-
sl-g (µm)	-	-	400	-	-	-	-
∑points_perikarya_	-	-	305	-	-	-	-
∑points_neuropil_	-	-	526	-	-	-	-

Details for stereological analysis of the corpus callosum (CC), cerebral cortex (CTX), subcortical area (SCTX), thalamus (TH), hypothalamus (HTH), basal ganglia (BG), and internal capsule (IC): sl-g = side length in *x-* and *y*-directions of the grids used to determine the volume; ∑points = mean number of counted points per animal; sl-uvf = side length in *x-* and *y*-directions of unbiased counting frames; B and H = base and height of the unbiased virtual counting spaces; D = distances between the unbiased virtual counting spaces in in *x-* and *y*-directions; ∑UVCS = mean number of unbiased virtual counting spaces per animal; ∑neurons = mean number of counted neurons per animal.
